# Structure and Mechanics of Supporting Cells in the Guinea Pig Organ of Corti

**DOI:** 10.1371/journal.pone.0049338

**Published:** 2012-11-07

**Authors:** Deborah E. Zetes, Jason A. Tolomeo, Matthew C. Holley

**Affiliations:** Department of Biomedical Science, University of Sheffield, Sheffield, United Kingdom; University of Washington, Institute for Stem Cells and Regenerative Medicine, United States of America

## Abstract

The mechanical properties of the mammalian organ of Corti determine its sensitivity to sound frequency and intensity, and the structure of supporting cells changes progressively with frequency along the cochlea. From the apex (low frequency) to the base (high frequency) of the guinea pig cochlea inner pillar cells decrease in length incrementally from 75–55 µm whilst the number of axial microtubules increases from 1,300–2,100. The respective values for outer pillar cells are 120–65 µm and 1,500–3,000. This correlates with a progressive decrease in the length of the outer hair cells from >100 µm to 20 µm. Deiters'cell bodies vary from 60–50 µm long with relatively little change in microtubule number. Their phalangeal processes reflect the lengths of outer hair cells but their microtubule numbers do not change systematically. Correlations between cell length, microtubule number and cochlear location are poor below 1 kHz. Cell stiffness was estimated from direct mechanical measurements made previously from isolated inner and outer pillar cells. We estimate that between 200 Hz and 20 kHz axial stiffness, bending stiffness and buckling limits increase, respectively,∼3, 6 and 4 fold for outer pillar cells, ∼2, 3 and 2.5 fold for inner pillar cells and ∼7, 20 and 24 fold for the phalangeal processes of Deiters'cells. There was little change in the Deiters'cell bodies for any parameter. Compensating for effective cell length the pillar cells are likely to be considerably stiffer than Deiters'cells with buckling limits 10–40 times greater. These data show a clear relationship between cell mechanics and frequency. However, measurements from single cells alone are insufficient and they must be combined with more accurate details of how the multicellular architecture influences the mechanical properties of the whole organ.

## Introduction

The mammalian organ of Corti is an elongated sensory epithelium that lies within the cochlea and that is adapted for the detection, amplification, and analysis of sound [1]. It is based upon a remarkable cellular architecture composed of several morphologically distinct types of sensory hair cells and supporting cells, each with specific dimensions and cytoskeletal specializations that change progressively from the apical, low frequency end of the cochlea to the basal, high frequency end [2], [3], [4], [5], [6]. This implies a close relationship between frequency tuning and the structure and mechanical properties of individual cells [7]. Accurate characterization of the mechanical properties of individual cells within the organ of Corti should thus help in the construction of more accurate models of cochlear mechanics [1]. Whilst attention has been given to the mechanical properties of hair cells [8], [9], [10], [11], [12], [13], [14], [15], [16] and their mechanosensory bundles [17], [18], a systematic analysis of supporting cells has not been undertaken.

**Figure 1:**
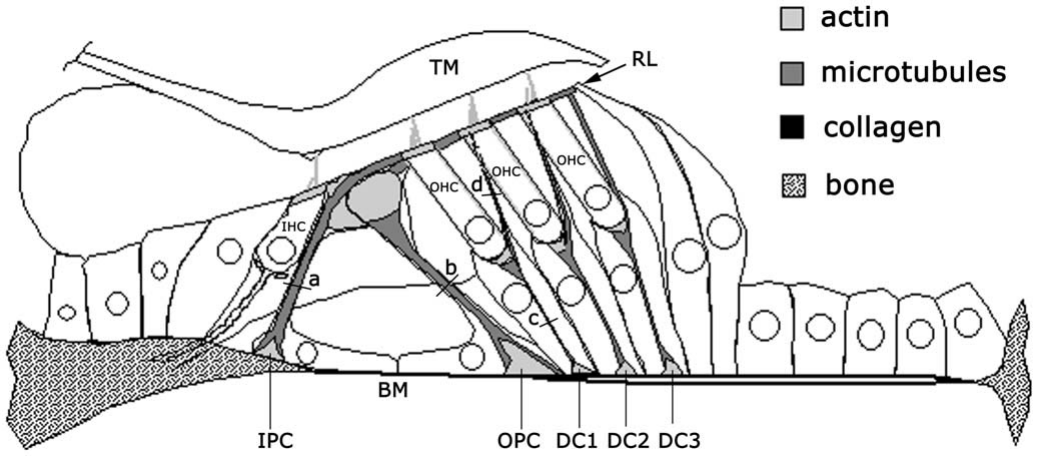
Diagrammatic cross-section of the organ of Corti to illustrate main cytoskeletal components. Structures containing actin filaments and microtubules are lightly and darkly shaded, respectively. Numbers of microtubules were counted from transverse sections at 4 different levels: a) the axial region of inner pillar cells (IPC) b) the axial region of outer pillar cells (OPC) c) the basal part of each row of Deiters' cells (DC1–3) d) the phalangeal processes of each row of Deiters' cells. The bases and apices of both pillar cells and Deiters' cells included dense actin meshes into which the microtubules were embedded. The axial regions of all cells were composed of parallel arrays of both microtubules and actin filaments. IHC – inner hair cell, OHC - outer hair cells, RL – reticular lamina, BM – basilar membrane, TM – tectorial membrane, a-d – levels of section illustrated in figure 2.

The organ of Corti normally includes a single row of inner hair cells and three rows of outer hair cells coupled to the basilar membrane by supporting cells (Fig. 1). The row of inner and first row of outer hair cells are separated by rows of inner and outer pillar cells, which form the arch or tunnel of Corti. Each row of outer hair cells is supported by a row of Deiters'cells. Unlike the hair cells, the bases and apices of all pillar cells and Deiters' cells span the whole sensory epithelium from the basilar membrane to the reticular lamina. Thus their lengths define the key structural dimensions of the sensory epithelium.

Each cell type within the organ of Corti has a characteristic cytoskeletal architecture that is defined by different arrangements of actin filaments and microtubules. In general terms, the mechanically dominant cytoskeletal component in hair cells is the actin filament, which takes a variety of cross-linked patterns to shape the cell body, apical cuticular plate and sensory hair bundle [19], [20]. In contrast, pillar cells and Deiters' cells are dominated by long bundles of microtubules interdigitated with actin filaments [3], [21], [22]. Qualitative observations show that at the high frequency end of the cochlea the number of microtubules is greater whilst the cells are shorter than they are at the low frequency end [21], [23], [24]. Stiffness measurements from intact and chemically extracted, dissociated outer pillar cells have been used to estimate the material properties of individual cells in the context of their cross-linked microtubule bundles [25]. This information has then been used to estimate stiffness values for Deiters' cells, which are difficult to dissociate intact [26]. There have been other estimates for Deiters' cells [27] but despite an extensive ultrastructural literature, few studies provide systematic data for changes with respect to each cell throughout the full length of the cochlea, making it difficult to relate mechanical properties at the cellular level to whole cochlear function.

In this study, we provide estimates of stiffness for pillar cells and Deiters' cells along the guinea pig organ of Corti. We assumed, as demonstrated both theoretically and experimentally [25], that stiffness can be calculated primarily from the number and length of microtubules in each cell type. Thus we made systematic measurements of these variables as a function of location along the organ of Corti, which can be correlated directly with frequency. The measurements were then used to estimate gradients of extensional and bending stiffness of supporting cells to provide baseline data for cellular models of cochlear mechanics.

## Materials and Methods

### Animal husbandry

Animal care and use was in accordance with the UK Home Office (Animal Procedures) Act 1986. Albino, Dunkin Hartley guinea pigs (appro×450 g) from Harlan Laboratories were killed by cervical dislocation before dissection of cochlear tissue, following procedures approved by the Ethical Review Committee at the University of Bristol, where the tissue was prepared.

### Dissociation of intact cells

Inner pillar cells, outer pillar cells and Deiters' cells were dissociated enzymatically and mechanically *in vitro* from two guinea pigs for light microscopy as previously described [25]. Exclusion of rhodamine-labeled phalloidin was used to confirm that the cell membranes were intact. Measurements from cells under these conditions were correlated with those from tissue prepared for electron microscopy.

### Electron microscopy

Six cochleae from three guinea pigs were prepared for transmission electron microscopy. Each cochlea was partially opened at each turn to expose the organ of Corti and the whole temporal bone was then fixed for 90 minutes with 2% ultra-pure glutaraldehyde in 0.1 M phosphate buffer pH 7.4 containing 1% tannic acid. Fixative was circulated through the cochlear duct with a pipette every 15 minutes. The temporal bone was then removed, and the cochleae were washed in 0.1 M phosphate buffer every 5 minutes for 30 minutes, post-fixed in 1% OsO_4_ in the same buffer for 90 minutes, dehydrated via a series of ethanols, infiltrated in Epon resin, and polymerized for 48 hours at 60° C. Each cochlea was cut into eight hemicoils (2 halves of each of the 4 turns) with a 0.15 mm thick radial diamond wafer blade (cut size 0.16±0.01 mm). The hemicoils and remaining hook region segments were photographed under conventional bright field light microscopy for measurement of the length of the organ of Corti and then re-embedded for further sectioning.

The six cochleae were separated into sets of left and right ears. Thick sections (2 µm) were cut in the radial plane from each hemicoil of the left ear set, stained with Toluidine Blue, and viewed in conventional light microscopy for measurement of pillar cells, Deiters' cells, outer hair cells, and the reticular lamina. Ultrathin sections (80 nm) were collected from hemicoils of the right ear set for measurement of small structures and for counting the microtubules. Specifically, seven hemicoils dispersed throughout the length of the cochlea across all three animals were sectioned in the transverse plane parallel to the sensory epithelial surface, which is also known as the reticular lamina. Two hemicoils were sectioned in each of the radial and circumferential planes. Sections were collected onto copper grids, stained with uranyl acetate and lead citrate, and viewed with a Philips CM100 transmission electron microscope. After sectioning, the lengths of the hemicoils were re-measured to estimate the precise location along the cochlea from which the thick and ultrathin sections had been taken. This estimate of position was used to obtain an estimate of the corresponding best frequency of the sectioned material from Greenwood's frequency-place map *f*  = 3500*(10^0.021*×*^- 0.85), where *f* is the characteristic frequency in Hz and *x* is percent distance along the guinea pig cochlear duct [28].

### Morphological measurement

Measurements were obtained from resin-embedded samples at both the light and electron microscope levels. Thick resin sections were viewed through a Nikon Diaphot inverted microscope. Dimensions of the pillar cells, hair cells, Deiters' cells, and basilar membrane were measured through video images processed with an image enhancer and analysis software (Brian Reece Scientific Ltd). Dimensional measurements of the arch of Corti were taken along the central axes of the inner pillar cells and outer pillar cells from their apices to their apposition with the basilar membrane. The basal separation between the pillar cells was obtained between their central axes in the plane of the basilar membrane. Hair cell lengths were measured from the upper edge of the cuticular plate to the base. The length of the basal part of each Deiters' cell was taken from the base of each outer hair cell to the intersection of the Deiters' cell axis with the basilar membrane. The width of the basilar membrane, including both the arcuate and pectinate zones, was determined between the narrowest point of the bony shelf and the spiral limbus. At least 3 sections and 6–10 measurements were obtained from the 3 different cochleae for each dimension. Quantification of uncertainties from nonsystematic errors was obtained by repeated measurements of each structure at each magnification. Additional sections taken in the orthogonal direction illustrated negligible compression of the material in the sectioning process. In ultrathin sections, microtubules were counted from the central regions of inner pillar cells, outer pillar cells and all three rows of Deiters' cells and their processes at 4–5 locations along the cochlear duct in each animal.

## Results

### Supporting cell morphology

**Figure 2:**
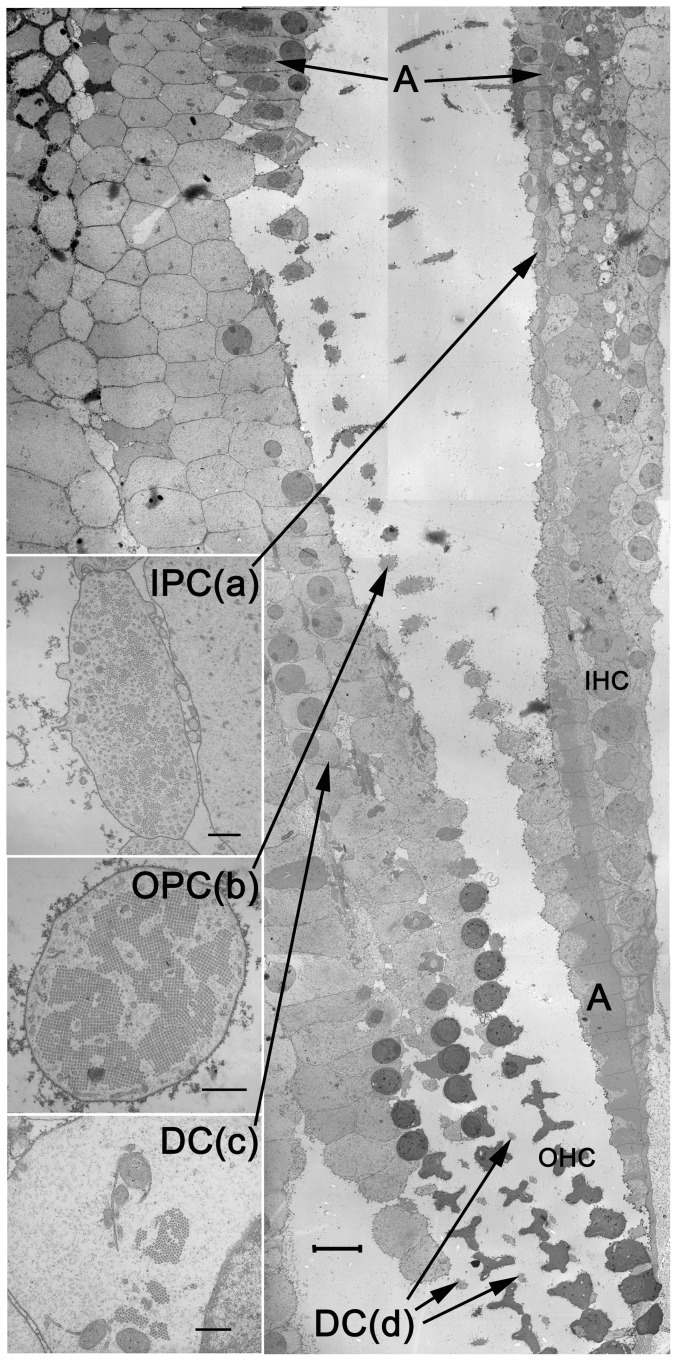
Electron micrograph of a section cut in the same plane as the basilar membrane. The section passes at a slightly oblique angle to cut supporting cells transversely across their axes at different levels along the organ of Corti between the basilar membrane and the reticular lamina. The insets are representative of the regions to which they refer.IPC(a) – inner pillar cell at level equivalent to that labeled a in figure 1 OPC(b) – outer pillar cell at level equivalent to that labeled b in figure 1 DC(c) – Deiters' cell at level equivalent to that labeled c in figure 1 DC(d) – Deiters' cell phalangeal process at level equivalent to that labeled d in figure 1 Note that inner pillar cell shafts are oval in cross-section and lie adjacent to the inner hair cells, that outer pillar cell shafts are rounded in cross-section with no mechanical support from adjacent cells, that the basal portion of Deiters' cells contain relatively small bundles of microtubules with a much higher proportion of cytoplasm than that seen in PCs, and that the phalangeal processes of Deiters' cells are thin and rounded in cross-section with no mechanical support from adjacent cells. A - Dense actin networks at the bases and apices of the pillar cells IHC – inner hair cells OHC – outer hair cells (distorted during preparation) Scale bar = 10 µ (insets = 500 nm).

Low magnification images were taken from ultrathin sections on coated, slotted grids to allow the individual cells and levels of section illustrated in Figure 1 to be identified at higher magnification (Fig. 2).

#### Pillar cells

The cross-sectional profiles and organization of microtubules differed both along and between inner pillar cells and outer pillar cells. The profiles of inner pillar cell shafts were oval as if flattened against the adjacent rows of inner hair cells and their associated inner phalangeal cells, and each shaft was in contact with the neighbouring inner pillar cells via the lateral membrane (Fig. 2a). The axial microtubules were loosely bundled and where they were cross-linked together they formed hexagonal arrays in which each microtubule was surrounded by about 6 actin filaments. By contrast, the shafts of outer pillar cells were narrower and rounded in cross-section with the plasma membrane enclosing a more tightly bundled array of microtubules (Fig. 2b). The axial microtubules were much more densely packed and often in square arrays in which each microtubule were surrounded by only 4 actin filaments, as illustrated previously [25]. At the base of both types of pillar cell the microtubule bundles splayed outward and terminated in a footplate of densely-packed actin filaments, which normally sits directly on top of the basilar membrane.

The apical regions were similar in design to a hinge joint in which the convex apical profile of the outer pillar cell was inserted into a concave recess in the adjacent inner pillar cell. A slender microtubular process projected from the top of the outer pillar cell and was attached to the second row of outer hair cells. The pillar cell nuclei were located basally and inside the floor of the tunnel of Corti (Fig. 1).

Serial, transverse sections through outer pillar cells were correlated with their profiles. Outer pillar cells were divided into 3 regions of approximately equal length, namely apical and basal cones joined by a narrow shaft. The arrangement of microtubules changed dramatically through these regions. In the base of the cell at the level of the nucleus many microtubules were single or deployed in loose bundles of 5–50. These microtubules were distributed both within and around the actin complex and they were separated by numerous membrane vesicles and mitochondria. Moving towards the lower region of the shaft the microtubule bundles became fewer and larger. Only in the middle region of the shaft, equivalent to about one quarter of the total cell length, were the bundles tightly packed into a square array. Towards the upper region of the shaft the bundles splayed out and into the apical cone. The apical cone overlapped more or less completely with the adjacent outer hair cells, which meant that the shafts of the outer pillar cells were aligned in parallel with the bodies of the Deiters' cells (Fig. 2).

#### Deiters Cells

The three rows of Deiters' cells support the three rows of outer hair cells and the axis of the slender bundle of microtubules in the basal part of each cell was approximately aligned with that of the associated outer hair cell (Fig. 1). A phalangeal process from this bundle extended beyond the base of the outer hair cell and up to the reticular lamina at the epithelial surface. Processes from the first and second rows of Deiters' cells separated the second and third row outer hair cells from their neighbours and those from the third row of Deiters' cells provided a border along the outer edge of the third row of outer hair cells. The phalangeal processes, like the outer pillar cell shafts, were rounded in cross-section and without contact with adjacent cells (DC(d) in Fig. 2). However, whilst the Deiters' cell bodies were aligned in parallel with outer pillar cell shafts the phalangeal processes were aligned more or less in parallel with the outer hair cells.

### Cell length

**Figure 3:**
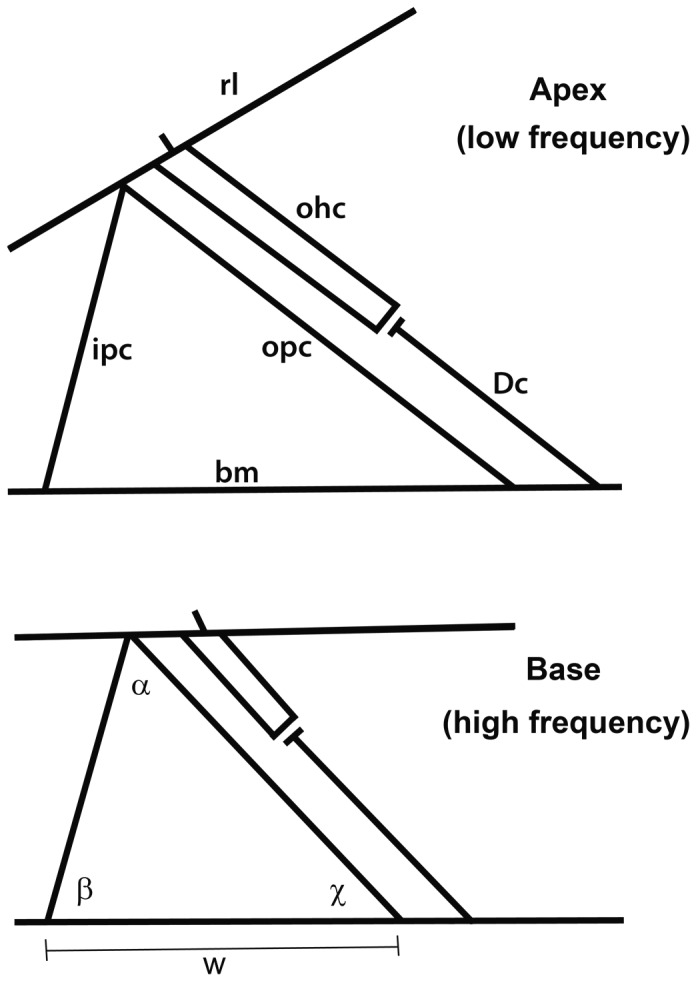
Diagram of the key measurements made from the organ of Corti to illustrate the differences between dimensions at the base and apex of the cochlea. The profiles illustrated in this figure were taken from histological sections cut near to the apex and close to the base of a guinea pig cochlea. The profile from the apex is labeled to indicate the basilar membrane (bm), reticular lamina (rl), inner and outer pillar cells (ipc, opc), a single Deiters' cell (Dc) and a single outer hair cell (ohc). The lower profile from the base illustrates the angle between the upper ends of ipc and opc (α), and inside the arch of Corti between the ipc and bm (β) and between the opc and the bm (χ). The distance w defines the separation between the bottom ends of the ipc and opc. Note that the greatest measured changes in cell length were in the ohc and opc with the length of the Dc remaining almost constant. Whilst w decreased towards the base the associated changes in cell length meant that α remained almost constant.

**Figure 4:**
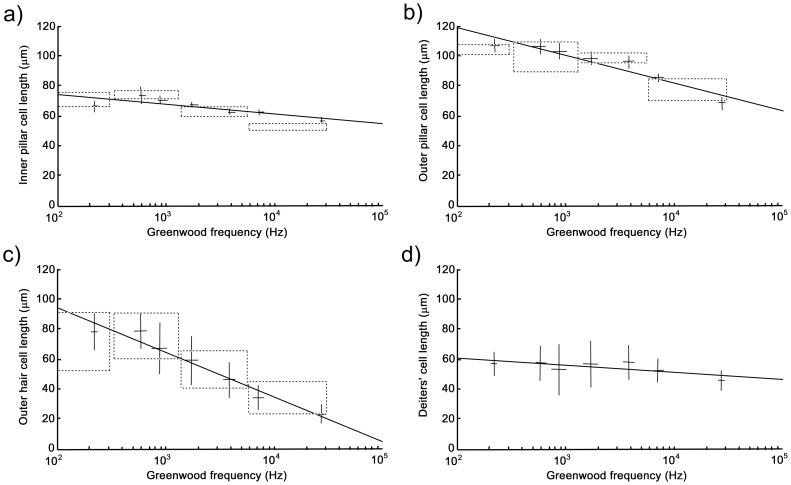
Cell length measurements plotted against Greenwood frequency along the cochlear duct. The mean length of inner pillar cells (a) decreased towards the basal high frequency end of the cochlea and a similar but more pronounced decrease was measured for outer pillar cells (b). The arch of Corti was consequently smaller at the high frequency end of the cochlea. The mean length of the outer hair cells decreased more dramatically (c) whilst there was only a relatively small decrease in the mean length of the Deiters' cell bodies (d). Dashed boxes indicate verification of measurements obtained from dissociated cells from individual cochlear turns. Lines are linear regression fits to raw data. Error bars are two standard deviations about the mean. All correlation coefficients are highly significant. Mean values were derived from three animals.

Cell lengths were measured at seven frequency locations along the cochlear duct. The progressive changes are illustrated in diagrammatic sections taken from the apex and base of the guinea pig cochlea (Fig. 3). All cell types became progressively shorter towards the base so that length was inversely correlated with frequency (Fig. 4). Inner pillar cells ranged from 75–55 µm long (Fig. 4a) whilst outer pillar cells were 120–65 µm long (Fig. 4b). The arch of Corti, which is defined by these two cell types, decreased in width towards the base of the cochlea. The length of inner pillar cells versus the log of Greenwood's frequency was fitted with a linear line of slope −6.56 with a significant correlation coefficient of 0.82. For outer pillar cells the slope was −18.53 with a correlation coefficient of 0.95. The separation between the bases of inner pillar cells and outer pillar cells also decreased towards the base, with a slope of −15.33 and a correlation coefficient of 0.90. This variation in arch geometry is such that the angle between the inner pillar cells and outer pillar cells is constant (Fig. 3). The angle inside the arch of Corti between the inner pillar cells and basilar membrane decreased slightly toward the base of the cochlea, whilst the adjacent angle between the outer pillar cells and basilar membrane increased.

The most striking changes in cell length were in the outer pillar cells and outer hair cells (Fig. 3). The outer pillar cells were nearly half as long at the base of the cochlea compared with the apex but the range for outer hair cells was much greater at ∼100–20 µm (Fig. 4c). The average length of the three rows of outer hair cells at a given location decreased linearly towards the base of the cochlea and was fitted versus Greenwood frequency with a slope of −29.15 and a correlation coefficient of 0.98. On average, outer hair cells in the first row were 20% shorter than those in the second row, which were 20% shorter than those in the third row. The mean measurement of the 3 rows was used for the correlation shown in Figure 4c. The average length of inner hair cells was 34.0 µm (SD = 4.4, n  = 31) and there was no significant, systematic change along the cochlea. This correlates with the relatively small change in the length of the inner pillar cells and the height of the arch of Corti.

In contrast to the outer hair cells, the mean length of the bases of the Deiters' cells, which defined the distances between outer hair cells and the basilar membrane, showed only a very small decrease from 60–50 µm (Fig. 4d). The slope was only −4.4 and the intercept was 68.9 µm, with a significant correlation coefficient of 0.7. The radial increase in the length of the outer hair cells from rows 1-3 is consistent with the divergent angle between the reticular lamina and basilar membrane and there is only a small radial variation in the length of the associated Deiters' cell bases. The lengths of the Deiters' cell phalangeal processes were not measured but they are very similar to those of the adjacent outer hair cells. However, they are tilted at an angle of up to 35° to the hair cell axes, which according to the cosine rule means that they are about 10% longer.

Measurements of outer hair cells, inner pillar cells and outer pillar cells from embedded sections were verified in vitro. The dashed boxes in Figure 4a–c indicate measurements obtained from light microscopy of dissociated cells from individual cochlear turns. This was not possible for the Deiters' cells as their bases did not retain their original shape in vitro.

In each set of measurements there was a plateau in the slope below 10^3^ Hz. This correlated with rough measurements of cell length from a virtual histological section of a guinea pig cochlea (http://www.med.umich.edu/histology/cns/ear.html#80) although in the latter there was a more pronounced decrease at the apex.

### Numbers of microtubules

**Figure 5:**
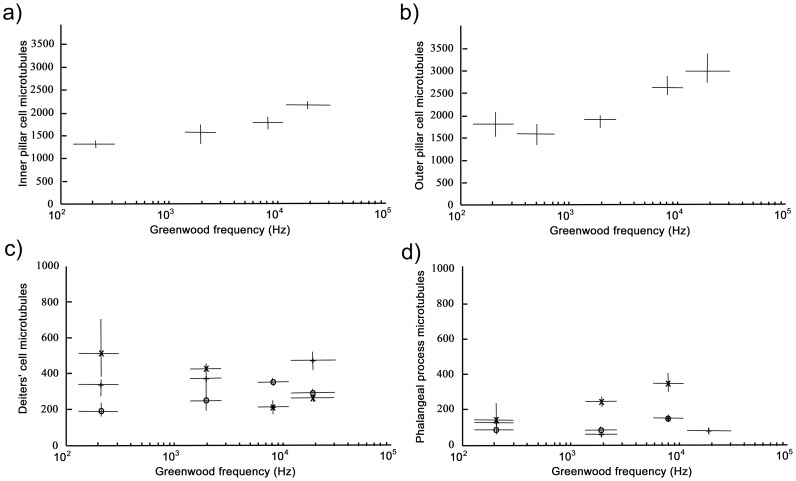
Number of microtubules plotted against Greenwood frequency along the cochlear duct. Inner (a) and outer (b) pillar cells contained more microtubules in the basal, higher frequency regions. There was no obvious relationship with frequency for numbers of microtubules in the Deiters' cell bodies (c) or phalangeal processes (d). Vertical and horizontal lines indicate measurement uncertainty as determined by two standard deviations about the mean from repeated measurement.

Microtubules are the dominant cytoskeletal structure within pillar cells and Deiters' cells. Thus they were cross-sectioned and counted from four to five locations along the cochlear duct (Fig. 5).

The number of microtubules increased in both inner pillar cells (Fig. 5a) and outer pillar cells (Fig. 5b) towards the basal, high frequency region of the cochlea with ranges of 1,300–2,100 and 1,500–3,000, respectively. Thus pillar cells became progressively shorter and thicker towards the base of the cochlea. Deiters' cells contained many fewer microtubules but the counts were variable with no clear relationship to location. The numbers in the cell body, below the OHCs, varied from 200–500 (Fig. 5c) whilst in the phalangeal processes it was from 30–300 (Fig. 5d). There was some evidence for a moderate increase in numbers for the second and third rows of Deiters' cells and a moderate decrease for the first row. Interestingly, the correlation between the numbers of microtubules and cochlear location for both inner and outer pillar cells was flatter or even reversed below 10^3^ Hz. This trend reflected the poor correlation between cell length and cochlear location in the same region (Fig. 4).

### Mechanical properties of supporting cells

The dominant structural components that define the mechanical properties of outer pillar cells are the length and number of microtubules. These parameters have previously been modeled in conjunction with direct experimental measurements from intact, isolated pillar cells [25] and they can be used to estimate the axial stiffness, bending stiffness and buckling limits of all pillar cells and Deiters' cells (Table 1). The experimental measurements were derived from preparations in which cells were fixed at both ends whilst a calibrated probe of known stiffness was used to displace the mid-point of the cell. The resultant measurement for bending stiffness was then used to derive axial stiffness and buckling limits. The axial or extensional stiffness is a measure of the resistance to elongation under tension along the length of the cell. The buckling limit is the force required to deform or to bend a cell under axial compression.

**Table 1:** Axial, bending, and buckling stiffness of supporting cells at selected frequency regions of the cochlea.

	Frequency			
	200 Hz	2000 Hz	8000 Hz	20000 Hz
	Axial stiffness (N/m)			
Outer pillar cell	1.90E+01	3.30E+01	4.40E+01	5.30E+01
Inner pillar cell	2.50E+01	3.60E+01	4.40E+01	5.00E+01
Deiters' cell body	8.50E+00	8.70E+00	8.90E+00	9.00E+00
Deiters' cell phalanx	1.90E+00	4.50E+00	8.10E+00	1.30E+01

	Bending stiffness (Nm/m)			
Outer pillar cell	2.50E-07	6.10E-07	1.10E-06	1.50E-06
Inner pillar cell	8.00E-07	1.40E-06	1.90E-06	2.40E-06
Deiters' cell body	4.20E-07	5.00E-07	5.60E-07	6.00E-07
Deiters' cell phalanx	1.70E-07	4.90E-07	1.50E-06	3.50E-06

	Buckling limit (N)			
Outer pillar cell	9.40E-11	1.90E-10	2.90E-10	3.90E-10
Inner pillar cell	1.90E-10	3.00E-10	3.90E-10	4.70E-10
Deiters' cell body	8.10E-11	8.90E-11	9.50E-11	9.90E-11
Deiters' cell phalanx	1.20E-11	4.60E-11	1.20E-10	2.90E-10

#### Axial Stiffness

Most models of the organ of Corti use end force to end displacement stiffness as a parameter. For a cell with microtubules along its length, the extensional stiffness may be written
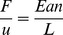



Where *F* is axial force, *u* is the axial deflection, *L* is length of the cells (also the microtubules), *E* is the Young's modulus of a single microtubule, *a* is the cross-sectional area of a single microtubule and *n* is the total number of microtubules. In the case of the pillar cells and Deiters' cells, the contribution of the apical and basal actin caps into which the microtubules are embedded is negligible. Actin has a Young's modulus of similar magnitude to microtubules, and the caps have a much larger cross-sectional area than the thin middle region of the cells. The actin caps therefore appear rigid in comparison to the microtubules, such that the compliance of the microtubules dominates.

#### Bending Stiffness

The bending stiffness of a structure is generally defined as the tip displacement for an applied out-of-plane load. A complete description of bending stiffness often includes a description of the structure's flexural rigidity, or product of the Young's modulus and the area moment of inertia. In the case of microtubule bundles, the flexural rigidity may be approximated by the Young's modulus and the sum of the moments of inertia of microtubules. For an out-of-plane load, the bending stiffness of a microtubule bundle is given by
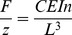
where *F* is the magnitude of the out-of-plane load, *z* is the tip displacement, *L* is the length of the cell, *I* is the moment of inertia of a single microtubule, *E* is the Young's modulus of a single microtubule, *n* is the total number of microtubules along the cell length, and C is a coefficient which depends on the boundary conditions. For the case of an end-loaded cantilever with one end fixed and the other free, *C* = 3.

#### Buckling Limit

The buckling load limit for each cell type was calculated from the bending stiffness, length, and end conditions of the cell. For comparative purposes it was assumed that both ends of the cell were simply supported.
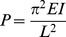



The stiffnesses and load carrying capabilities of each cell type were calculated from the data illustrated in Figures 4–5 and are presented in Table 1. We assumed that the Deiters' cell phalangeal processes had similar lengths to their adjacent outer hair cells. In fact they are likely to be slightly longer because their bases are attached to outer hair cells some 3-4 cells away from those of their apices.

### Relative properties of supporting cells

**Figure 6:**
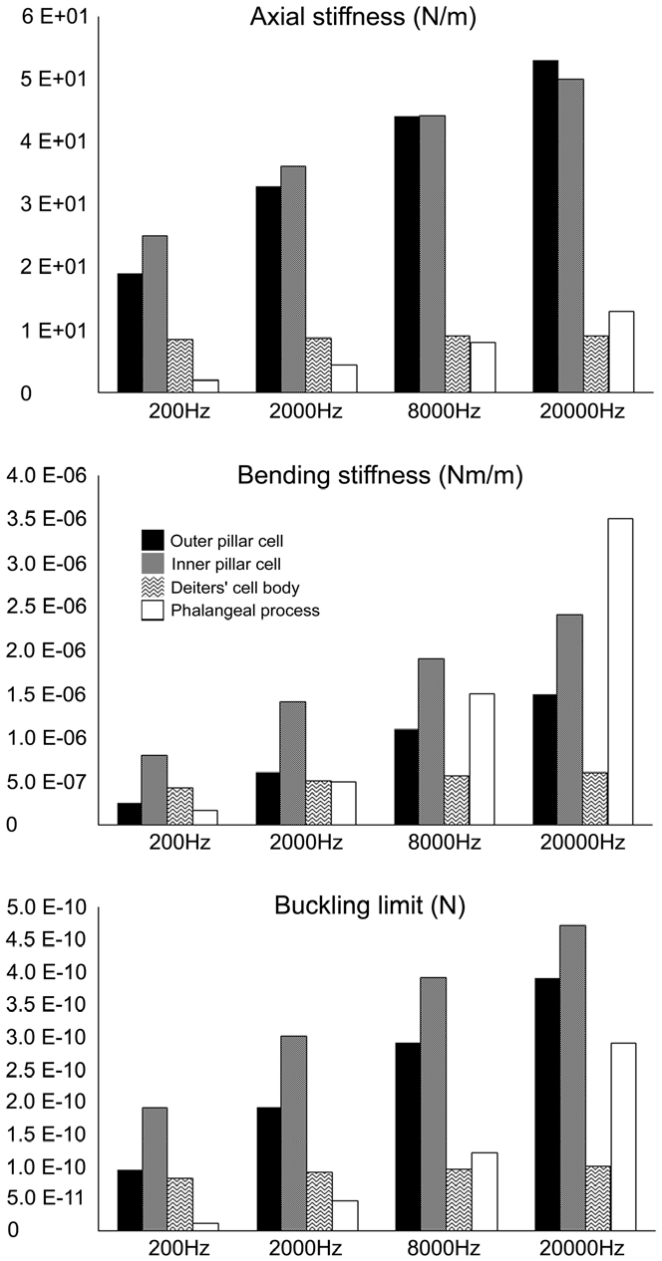
The data from **Table 1** plotted to show relative stiffness values between cells.

The data in Table 1 reveals the relationship between the material properties of the supporting cells and their frequency location (Fig. 6). Axial stiffness, bending stiffness and buckling limits generally increase towards the higher frequency regions of the cochlea. Nevertheless, whilst outer hair cells vary in length from over 100 µm to less than 20 µm from apex to base, the bodies of the Deiters' cells, which define the distance between the bases of the outer hair cells and the basilar membrane, changes from only ∼60–50 µm. There are only 200–500 microtubules in these cells and there is no clear, systematic change in number along the cochlea. Thus it seems that all outer hair cells, independent of location, are coupled to the basilar membrane by Deiters' cell structures of very similar mechanical properties. This is reflected in our estimates of axial stiffness, bending stiffness and buckling limit (Table 1). The phalangeal processes project from the bases of the outer hair cells to the reticular lamina and thus vary in length to a similar extent. They rarely have more than 200 microtubules and there is little correlation with location along the cochlea, save perhaps for the first row of Deiters' cells in which the number increases around 2–3 fold (Fig. 5d). However, length is the dominant value in terms of stiffness, which means that axial stiffness, bending stiffness and buckling limit of the phalangeal processes increases from the locations equivalent to 200 Hz to 20,000 Hz by factors of about 7, 20 and 24, respectively (Table 1). The equivalent change in these values for the pillar cells is smaller but significant with ∼3, ∼6 and ∼4 fold increases for outer pillar cells and ∼2, ∼3 and 2.5 fold increases for inner pillar cells.

Length is a critical factor in the models, which may explain why the bending stiffness of Deiters' cell phalangeal processes is relatively large compared to that of the pillar cells. This issue is considered in more detail in the discussion. The most obvious observations from the results are that stiffness values generally increase with frequency along the cochlea, with the greatest changes being in the Deiters' cell phalangeal processes and the smallest being in the Deiters' cell bodies.

## Discussion

The progressive decrease in the lengths of pillar cells and the increase in the numbers of microtubules from the apical, low frequency end of the cochlea to the basal, high frequency end has been reported previously [2], [3]. In this study we have made systematic measurements along the guinea pig cochlea to try to correlate these changes more precisely with frequency and to make estimates of stiffness. To achieve this we have applied a model based on direct mechanical measurements of pillar cells isolated from the guinea pig cochlea, incorporating the material properties of their microtubules and cross-links [25]. These direct measurements of intact, isolated cells should be robust and in this study they should allow us to gain valuable insight into the magnitude of the changes in material properties of single cells along the organ of Corti. However, there are several factors in our measurements that influence absolute estimates of cell stiffness. Furthermore, our interpretation of how these values influence cochlear function will depend on models that can represent the complex interactions and additional properties that exist across the networks of cells that make up the sensory epithelium.

### Cell length

**Figure 7:**
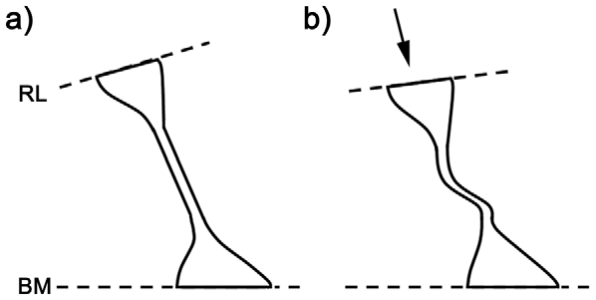
Structure and buckling of outer pillar cells. Outer pillar cells are normally straight (a) and span the space between the reticular lamina (RL) and the basilar membrane (BM). In many histological sections of the organ of Corti the central shaft is deformed (b) as if the structure has been subjected to a compressional force (arrow). Under these conditions the shapes of the actin-rich apical and basal cones remain unchanged (b). This implies that the cones are relatively stiff compared with the shaft and that the effective length of the cell is considerably shorter than the full length in terms of our microtubule model.

The estimated gradients in cell stiffness along the cochlea, as presented in the results, are likely to be accurate but the stiffness values should take account of a number of other aspects of cell structure. The first of these is the measurement of cell length and it is best illustrated through the analysis of longitudinal sections and serial, transverse sections. If the actin cones at the base and apex of each cell are considered to be solid, then the effective length measurement in terms of stiffness should be less than our cell length measurement L and may be as little as L/3 (Fig. 7). This view is reinforced by viewing almost any transverse section of the organ of Corti, where the middle section of the outer pillar cells is often buckled, whilst the extremities retain their shape. If effective values for L are one third of our measured values this would increase the axial, bending and buckling values by factors of 3, 27 and 9, respectively, making the bending stiffnesses of the pillar cells around 50–100 times greater than those of the Deiters' cell bodies. This would represent the upper limit for the pillar cells.

### Microtubule cross-linking

Cross-linking between microtubules also influences the bending stiffness. Direct measurements from intact and extracted outer pillar cells suggest that cross-links can increase stiffness by a factor of 4 [25]. Whilst we do not have specific measurements for cross-linking, the morphology suggests that it is greater in the outer pillar cells than in the inner pillar cells. This is because the microtubules in outer pillar cells are more closely bundled into square and hexagonal arrays (Fig. 2b). In inner pillar cells the microtubules are sparsely distributed (Fig. 2a), which implies that they are less tightly cross-linked. Our estimates, based on intact outer pillar cells, suggest that both cell types share similar stiffness values, with inner pillar cells being stiffer primarily because they are shorter. However, it is possible that lower levels of cross-linking mean that our estimates for the inner pillar cells are over-estimated by 2–3 fold, making the outer pillar cells the stiffest components of the organ of Corti.

### Buckling limits and morphology

The buckling limits for all supporting cells must also be influenced by their attachments to surrounding structures. Experimental measurements in this context are extremely difficult to make but consideration of microtubule bundles alone probably leads us to underestimate the functional stiffness of, for example, the Deiters' cell bodies. Cross-sections through the Deiters' cell bodies show that they are tightly packed to form a continuous layer (Fig. 2). If we imagine them to be hydraulic sacs containing a reasonably viscous cytoplasm then they may act as a relatively stiff layer. The microtubule bundles are surprisingly small to resist axial forces but they may function to define the space between outer hair cells and the basilar membrane. Inner pillar cells are supported by inner phalangeal cells and they are thus also likely to have a higher buckling limit than that estimated for isolated cells. Nam and Fettiplace (2010) described a finite element model of the mechanics of the organ of Corti but considered our experimental values for pillar cell stiffness [11] to be too low. The experimental values came from direct physiological measurements from intact and extracted cells, which should provide reasonably accurate estimates for single cells. However we know relatively little about the ways in which forces are distributed between cells across the architecture of the organ of Corti. The effective stiffness of cell layers, particularly in the closely packed, basal regions of Deiters' cells, is likely to be affected by cytoplasmic pressure and it could be significantly greater than estimates derived from the small bundles of microtubules. This kind of insight is likely to be required for realistic mechanical models based on experimental data from single cells.

It is worth noting that the most ordered bundles of microtubules are those within the outer pillar cell shafts and the phalangeal processes of the Deiters' cells, both of which are surrounded by perilymph and have no adjacent cells for support. Their cross-sectional profiles are round, whilst those of inner pillar cells are ovoid. These features could reflect adaptations to torsion in different cell types. A column with a symmetrical cross section is less likely to suffer torsional buckling before, or in combination with, lateral buckling, which may be important for cells that stand free within the perilymph. The organization of the inner pillar cells suggests that they provide a continuous wall of microtubules to strengthen the boundary between the inner hair cells and the tunnel of Corti. In cochlear models this might be included as a continuous structure rather than as a series of independent pillars, which applies more obviously to the outer pillar cells.

### Cell stiffness and frequency

The relationship between frequency and the material properties of supporting cells is apparent not only in terms of progressive changes along the cochlea but also through the loss of the correlation in the region below 1 kHz. In all of the measurements, whether from live or fixed tissue or from cell length or microtubule number, the relationship changes below this frequency (Fig. 4–5). The effect in terms of cell length can be measured from histological texts [29] and more easily from high resolution virtual histological sections (http://www.med.umich.edu/histology/cns/ear.html#cochlea). This discontinuity coincides with a clear change in the shape of the neuronal tuning curve [30]. Basilar membrane vibrations are less sharply tuned at the apex of the cochlea than at the base [1]. It has been suggested that apical outer hair cells contribute relatively little to the sensitivity of low-frequency hearing [31] and there is little evidence for mechanical non-linearity in rate-level functions for sensory nerve fibres [32] or from displacements of the cochlear partition [33] below 1 kHz. Thus the loss of a clear correlation between frequency and the structural features of the pillar cells is associated with a lower level of frequency tuning and sensitivity.

### Integrating cell measurements into cochlear models

The analyses described in this article have been limited to consideration of passive, quasi-static loads but it is more important to consider the coupled dynamic response of the organ of Corti as a whole. In particular, it is important to consider the viscous and inertial resistances of the endolymphatic and perilymphatic fluids. Although hydrodynamic effects will generally prevent sharp tuning of individual components, it seems only reasonable that the gradient in cell morphology and structure discussed here should extrapolate into a graded dynamic response. Measurements of the stiffness of the cochlear partition, a term that includes the basilar membrane, organ of Corti, and overlying tectorial membrane, generally consider "mechanical impedence" which includes load-specific stiffness, viscous resistance, and inertial effects. Measurements of the radial variation in stiffness of the cochlear partition suggest that pillar cells are an important structural element [34], [35], [36]. However, such measurements are insufficient to fully characterize the contributions of individual cells. Lack of quantitative data for morphological variations and stiffness descriptions of cytoskeletal elements along the cochlear duct has limited models in terms of providing accurate three dimensional modeling of the organ of Corti.

No experimental data is available for the reticular lamina, which is formed from the microtubular projections of pillar cells and Deiters' cells, and the cuticular or actin-based plates of the hair cells and pillar cells. These plates could be considered as nearly rigid inclusions surrounded by microtubule bundles that are aligned transversely across the organ. Systematic structural analysis of the microtubule bundles along the cochlea, similar to that explored in this paper, might thus provide some insight into any stiffness gradient in the reticular lamina that may correlate with frequency location.
